# Drawing structured plasmonic field with on-chip metalens

**DOI:** 10.1515/nanoph-2021-0308

**Published:** 2021-09-03

**Authors:** Yulong Wang, Changjun Min, Yuquan Zhang, Fu Feng, Guangyuan Si, Ling Li, Xiaocong Yuan

**Affiliations:** Nanophotonics Research Center, Shenzhen Key Laboratory of Micro-Scale Optical Information Technology, Shenzhen University, Shenzhen 518060, China; Songshan Lake Materials Laboratory, Dongguan 523808, China; Melbourne Centre for Nanofabrication, Victorian Node of the Australian National Fabrication Facility, Clayton, VIC, Australia

**Keywords:** bottle beams, plasmonic metalens, surface plasmon polariton

## Abstract

The ability to draw a structured surface plasmon polariton (SPP) field is an important step toward many new opportunities for a broad range of nanophotonic applications. Previous methods usually require complex experimental systems or holographic optimization algorithms that limit their practical applications. Here, we propose a simple method for flexible generation of structured SPP field with on-chip plasmonic metalenses. The metalens is composed of multiple plasmonic focusing nanostructures whose focal shape and position can be independently manipulated, and through their superposition, SPP fields with specially designed patterns are obtained. Based on this method, we demonstrate several structured SPP fields including S- and W-shaped SPP focal fields and tunable SPP bottle beams. This work could provide new ideas for on-chip manipulation of optical surface waves, and contribute to applications such as on-chip photonic information processing and integrated photonic circuits.

## 1 Introduction

Surface plasmon polariton (SPP), with the capability of breaking through the traditional optical diffraction limit [1, 2], shows advantages of nanoscale spatial resolution and tremendous localized electromagnetic field enhancement [3, 4]. It thus provides an effective tool for manipulating light in the deep sub-wavelength range and promotes a variety of applications including optical storage [5], super-resolution imaging [6, 7], optical sensing [8], optical tweezers [9], Raman scattering [10], [11], [12], on-chip photonic information processing [13], [14], [15], and others. With the rapid development of plasmonic photonic devices, how to precisely control the propagation of SPP in the near field to achieve desired structured plasmonic fields has become a research hotspot.

Currently, structured or complex SPP fields can be excited by two main methods. The first is to directly use structured light sources [9, 16], [17], [18], [19], such as vector beams [9, 16] and optical vortices [17, 18], to excite and generate the structured SPP fields, but usually requires a complex light field modulation system and thus not suitable for integrated devices. The second is to use holographic metallic nanostructures designed with different unit cells, such as nanograting [20], [21], [22], nanoslit [23], [24], [25], [26], [27], [28], nanoholes [29], [30], [31], however, complex optimization algorithms are required to generate the holographic structures [22, 25], [26], [27], [28], [29], [30], [31].

Here, we propose a new method to flexibly generate the desired structured SPP field with on-chip plasmonic metalenses. It is well known that an arc-shaped nanoslit on metal film can act as a plasmonic lens to excite and focus the SPP [24, 32], [33], [34], [35], [36], [37]. Thus, we could design different plasmonic lens structures to control the number, position, shape, and other characteristics of the generated SPP focus. By combining multiple plasmonic lenses with different structures to form plasmonic metalens, the corresponding different SPP focal fields can be superimposed to get the required structured SPP pattern. Based on this principle, we designed and generated a series of SPP fields, including S- and W-shaped SPP focal fields and SPP bottle beams, and the effectiveness of the proposed method is verified experimentally. This work greatly improves the degree of freedom for plasmonic field modulation, as well as provides new ideas for on-chip photonics information processing [14], and could have application potential in integrated nanocircuitry [38, 39], in-plane communications [40], and data processing [41].

## 2 Principle of the on-chip plasmonic metalens

**Figure 1:**
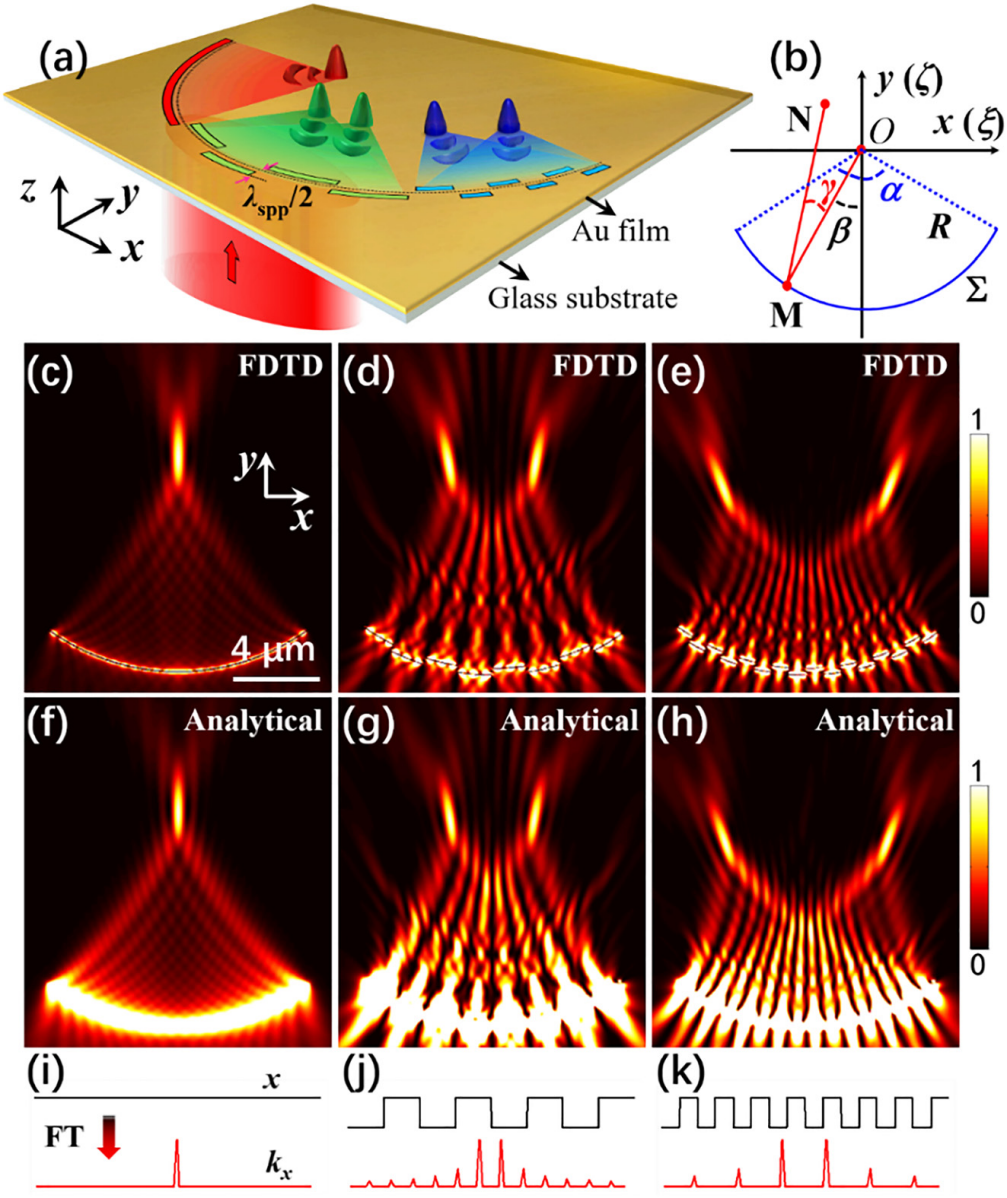
Principle of the on-chip plasmonic metalens. (a) Schematic diagram of the SPP focusing process excited by a plasmonic metalens structure, which is composed of a gold film (200 nm in thickness) etched with multiple discrete nanoslits (100 nm in width) around a circular reference arc (black dashed curve with a radius of *R*) on a glass substrate (*n* = 1.515). When illuminated with a laser (*λ* = 632.8 nm), the wavefront of SPP excited by the nanoslits is modulated by the displacement of slit position with respect to the reference arc (*R* = 10 μm), thereby realizing multi-focus modulation of SPP. (b) Schematics of in-plane SPP focusing. The field at any point **N**(*x*, *y*) in the vicinity of focus ***O*** (0, 0) can be calculated by summing up the contributions from all the points, e.g., **M**(*ξ*, *ζ*) on a convergent wavefront. *γ* measures the inclination angle of the distance with respect to the normal of the arc ∑. *β* represents the angle between **M*O*** and the *y*-axis. (c)–(e) The FDTD results of the SPP fields excited by plasmonic metalens structures in the red, green, and blue areas in (a), respectively, with the parameters: (c) *α* = 72°, (d) *α* = 72°, *p* = 18°, (e) *α* = 72°, *p* = 9°. (f)–(h) are the analytical calculation results corresponding to (c)–(e). (i)–(k) are schematic diagrams of FT of square waves for the three cases in (c)–(e), respectively.

Figure 1(a) shows the schematic diagram of the SPP focusing process excited by a plasmonic metalens structure, which is composed of a gold film etched with multiple discrete nanoslits around a circular reference arc (black dashed curve with a radius of *R*) on a glass substrate. Here, in order to avoid the directly transmitted light interfering with the excited SPP in the experiment, the thickness of the gold film is selected to be 200 nm, which is much larger than the penetration depth (about 28 nm) of the gold film. Since increasing the width of the slit could not only increase the excitation efficiency of SPP but also enhance the noise from the directly transmitted light through the slit, the width of the slit is chosen as 100 nm as a trade-off between the excitation efficiency of SPP and the signal-to-noise ratio in the experiment. The structure is normally illuminated by a plane-wave source from the bottom with a wavelength of *λ* = 632.8 nm. For a common plasmonic lens with a single arc nanoslit (red area in Figure 1(a)), SPP with converging wavefront is excited due to the scattering of the arc nanoslit, and then form a strong SPP focus at the geometric center. The corresponding three-dimensional finite-difference time-domain (3D-FDTD, Lumerical FDTD Solutions v8.19) simulation result is shown in Figure 1(c), which presents the focal spot and is consistent with the previous reports [32], [33], [34], [35], [36], [37]. When the single arc nanoslit structure is split into multiple discrete nanoslits (green area in Figure 1(a)) that forms a double-layer grating structure with a radial distance of *λ*_spp_/2 (*λ*_spp_ = 605.8 nm is the SPP wavelength), two closely spaced SPP focus near the geometric center are formed as shown in Figure 1(d). When reducing the angular period (*p*) of the grating (blue area in Figure 1(a)), the distance between the two SPP focus in the *x*-direction becomes larger as shown in Figure 1(e). We find that these dual-focus results in FDTD simulation can be explained by Fourier transform (FT) theory.

For the SPP excited by a common plasmonic lens, its dominated electric-field component ***E***_**z**_ perpendicular to the metal surface can be approximated as [13],

(1) $$E_{z}\left(x,y\right)\approx \frac{\sqrt{R}\exp\left(-ik_{\text{spp}}R\right)}{\pi }\int_{-\alpha /2}^{\alpha /2}\frac{\exp\left(ik_{\text{spp}}d\right)}{\sqrt{d}}U\left(\beta \right)d\beta$$

where, *k*_spp_ = 2π/*λ*_spp_ is the wave vector of SPP, *λ*_spp_ represents the wavelength of SPP [2, 42]. As indicated in Figure 1(b), *α* represent the maximal opening angle of the arc, *β* represents the angle between **M*O*** (the normal of the arc) and the *y* axis, *U*(*β*) is the complex amplitude at any point **M**(*ξ*, *ζ*) on the arc Σ, and *d* = [(*ξ* − *x*)^2^ + (*ζ* − *y*)^2^]^1/2^ is the distance between **M**(*ξ*, *ζ*) and the reference point **N**(*x*, *y*). The field at any point **N**(*x*, *y*) in the vicinity of focus ***O*** (0, 0) can be calculated by summing up the contributions from all the points, e.g., **M**(*ξ*, *ζ*) on a convergent SPP wavefront, as shown in Figure 1(b).

Using the following approximation:

(2) $$\begin{array}{l} d=\sqrt{{\left(\xi -x\right)}^{2}+{\left(\zeta -y\right)}^{2}}=\sqrt{R^{2}+{\text{x}}^{2}+y^{2}-2\left(\xi x+\zeta y\right)}\\ \approx R\sqrt{1-\frac{2\left(\xi x+\zeta y\right)}{R^{2}}}\approx R\left[1-\frac{1}{R^{2}}\left(\xi x+\zeta y\right)\right]\\ =R-\frac{\xi x+\zeta y}{R} \end{array}$$

We have:

(3) $$\begin{array}{l} E_{z}\left(x,y\right)\approx \int_{-\pi }^{\pi }\int_{0}^{\infty }U\left(\beta \right)\prod \left(\frac{\beta }{\alpha /2}\right)\delta \left(r-R\right)\exp\left[-\frac{ik_{spp}}{R}\left(\xi x+\zeta y\right)\right]rdrd\beta \\ \approx \int_{-\pi }^{\pi }\int_{0}^{\infty }U^{\prime }\left(\xi^{\prime },\zeta^{\prime }\right)\exp\left[-i2\pi \left(\xi^{\prime }x+\zeta^{\prime }y\right)\right]d\xi^{\prime }d\zeta^{\prime } \end{array}$$

where, *U′*(*ξ′*, *ζ′*) ≈ *U*(*β*)Π(2*β* **/** *α*)*δ*(*r* − *R*), *ξ′* = Re(*k*_spp_)*ξ* **/**(2π*R*) = *ξ* **/**(*Rλ*_spp_), and *ζ′* = Re(*k*_spp)_ *ζ/*(2π*R*) = *ζ* **/**(*Rλ*_spp)_, П(•) and *δ*(•) are the rectangular function and the Dirac delta function, respectively. Ignoring the constant term, Eq. (3) actually forms a two-dimensional (2D) FT relationship between the complex amplitude *U′*(*ξ′*, *ζ′*) along the arc and the ***E***_**z**_ component near its geometric focus as:

(4) $$E_{z}\left(x,y\right)=F_{2}\left\{U^{\prime }\left(\xi^{\prime },\zeta^{\prime }\right)\right\}$$

where, *F*_2_ denotes the 2D spatial FT.

By using the above equations, Figure 1(f)–(h) show the analytical calculation results corresponding to Figure 1(c)–(e). It can be clearly observed that the results obtained by these two methods are in good agreement, which verifies the correctness of the proposed analytical model. Moreover, in order to intuitively understand the reason why single focus (Figure 1(c) and (f)) or dual-focus (Figure 1(d), (e), (g), and (h)) could be generated by these plasmonic lens structures, we compared the FT effects of square wave functions with different periods, as shown in Figure 1(i)–(k). As is known that, the FT of a constant is the Dirac delta function (Figure 1(i)). As a result, the conventional plasmonic lens, whose phase distribution of excited SPP at the arc slit is a constant, can only focus the SPP at its geometric center and form a single focus (Figure 1(c) and (f)) corresponding to the Dirac delta function. For the double-layer grating structures (green area in Figure 1(a)), the phase distribution of SPP excited by the two-layer nanoslits likes a rectangular wave function. Since the rectangular wave can be considered as the superposition of countless sine/cosine functions, after being Fourier transformed, a positive and negative first-order spatial frequency shift occurs and forms two main peaks in the Fourier spectrum (Figure 1(j)), thus resulting in an SPP dual-focus (Figure 1(d) and (g)). When reducing the period of the rectangular wave (blue area in Figure 1(a)), the spatial frequency shift becomes larger (Figure 1(k)), leading to an increase in the distance between the dual-focus (Figure 1(e) and (h)).

With this FT theory, several specially distributed plasmonic fields, such as plasmonic Airy/Weber beams [13] and the multiple foci here, can be generated. However, since a single FT structure has to meet the paraxial approximation condition, and many complex 2D SPP patterns are difficult to be generated with a single FT function, which limits its application in the generation of complex SPP patterns. Therefore, we propose to combine multiple plasmonic lenses into metalens, to greatly enhance the ability to generate complex SPP fields.

## 3 Results and analysis

**Figure 2:**
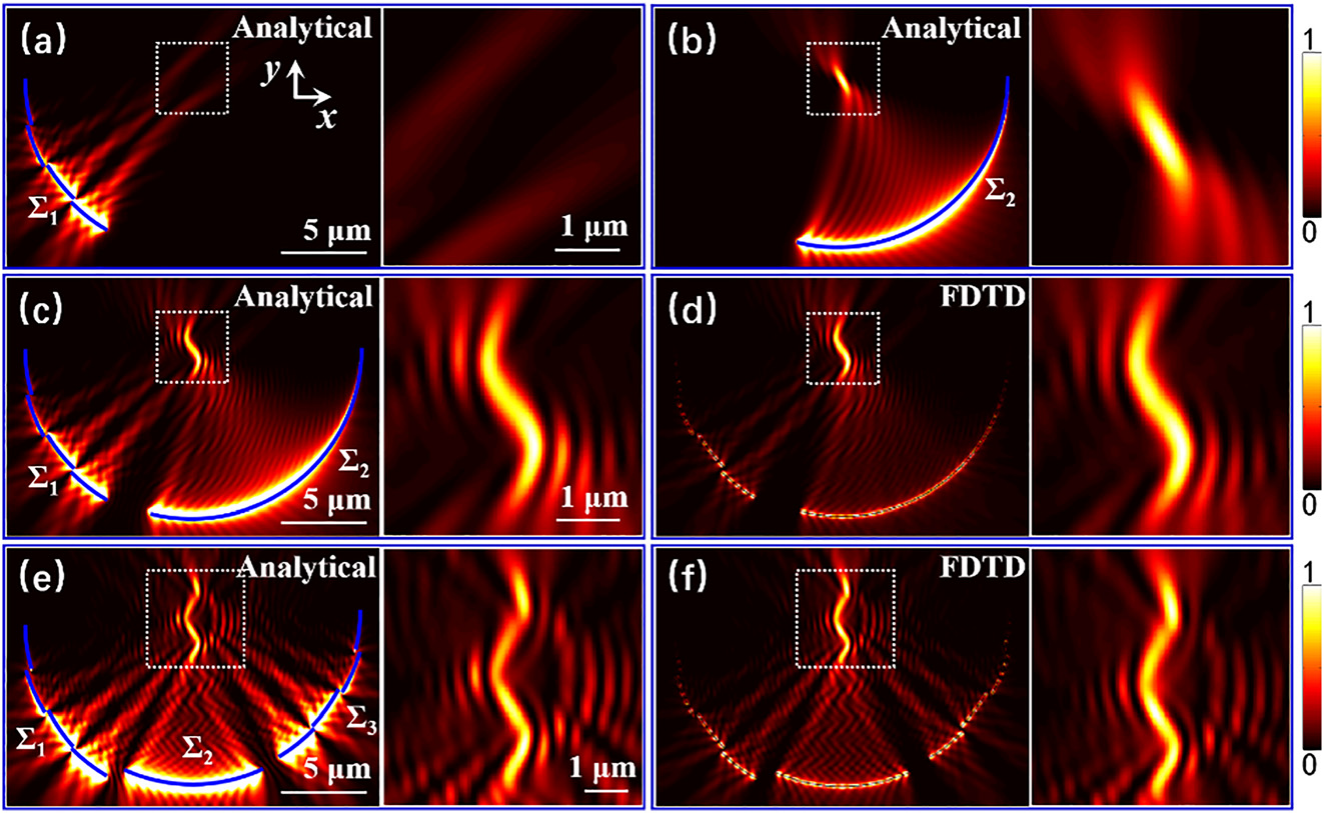
Structured SPP focal fields excited by plasmonic metalens structures marked by blue solid lines. (a) and (b) SPP field distribution excited by a plasmonic lens Σ_1_ and a plasmonic lens Σ_2_, respectively. The plasmonic lens Σ_1_ is a double-layer grating structure, whose radius of the inner and outer slits are *R* − *λ*_spp_/4 and *R* + *λ*_spp_/4 (*R* = 10 μm), respectively. And the angular period of Σ_1_ is *p* = 30°. The plasmonic lens Σ_2_ is a single slit with a radius of *R* − *λ*_spp_/4. For Σ_1_ and Σ_2_, the opening angles are *α* = 60° and 105°, respectively. (c) Analytical result and (d) FDTD simulation result of an S-shaped SPP field generated by a metalens composed of two plasmonic lenses Σ_1_ and Σ_2_. All parameters are as in (a) and (b). (e) Analytical result and (f) FDTD simulation result of a W-shaped SPP field generated by a metalens composed of three plasmonic lens Σ_1_, Σ_2_, and Σ_3_. The opening angles of Σ_1_, Σ_2_, and Σ_3_ are *α* = 60°, 48°, and 60°. For Σ_1_ and Σ_3_, the radius of the inner and outer arcs-shaped slits are *R* − *λ*_spp_/4 and *R* + *λ*_spp_/4, respectively, and the angular periods are both *p* = 30°. The radius of Σ_2_ is *R* − *λ*_spp_/4. To make the W-shaped SPP pattern more continuous, the structures Σ_1_ and Σ_3_ are shifted with a distance of −*λ*_spp_ and *λ*_spp_ in the *y*-direction, respectively. All other parameters are as in Figure 1.

Next, the above-mentioned plasmonic lens with controllable SPP focus is used to form the on-chip plasmonic metalens. Through the superposition of multiple SPP focal points, SPP fields with specially designed focal shapes or patterns can be obtained. Figure 2(a) and (b) show the SPP field distribution excited by a plasmonic lens Σ_1_ with multiple slits (indicated by blue arc segments) and another plasmonic lens Σ_2_ with a single slit, respectively. The plasmonic lens Σ_1_ generates SPP dual-focus tilted to the right around the geometric center, while the plasmonic lens Σ_2_ forms a single SPP focus tilted to the left at the center. When the two structures are combined into a plasmonic metalens structure, after the interference and superposition occurred between these SPP focuses, the final focal field appears as an S-shaped continuous pattern, as shown in Figure 2(c). It should be noted that the more complex the desired SPP pattern is, the more SPP focal points should be divided into, and thus more different plasmonic lenses are required. As shown in Figure 2(e), in order to form a W-shaped SPP pattern, a metalens structure composed of three different parts is used, where the SPP excited by the three plasmonic lens Σ_1_, Σ_2_, and Σ_3_ respectively forms: dual-focus tilted to the right, a single focus along the *y*-axis, and dual-focus tilted to the left in the central region. After the interference and superposition of these shifted foci, a W-shaped SPP focal field is achieved. It should be noted that here the structures Σ_1_ and Σ_3_ are shifted with a distance of −*λ*_spp_ and *λ*_spp_ in *y*-direction relative to the reference arc (black dashed curve in Figure 1(a)), respectively. These shifts are used to precisely control the relative position between multiple SPP focal points so that the generated W-shaped SPP pattern could be a continuous pattern rather than separate multiple focal points. The corresponding FDTD simulation results shown in Figure 2(d) and (f) are in good agreement with the analytical ones, proving the validity of the plasmonic metalens structures in the generation of the SPP focal field with a special shape or pattern.

It is well known that the method of plasmonic holograms can also be used to generate structured SPP fields. Holographic plasmonic structures with nanoholes or nanoslits can achieve a variety of modulation effects of SPP, such as focusing SPP and wavelength demultiplexing [43, 44], nonperfect Bragg diffractions [45, 46], and SPP bending [47]. Although this method has powerful modulation functions, however, it usually requires complex iteration and optimization algorithms to design the phase distribution of the lattice structure. Moreover, the lattice structure could generate strong scattering, and each nanohole/slit in the lattice could affect the propagation of SPP excited by the adjacent lattice structure. In contrast, our design method is much simpler based on the FT principle, and the designed structures could have lower scattering noise.

**Figure 3:**
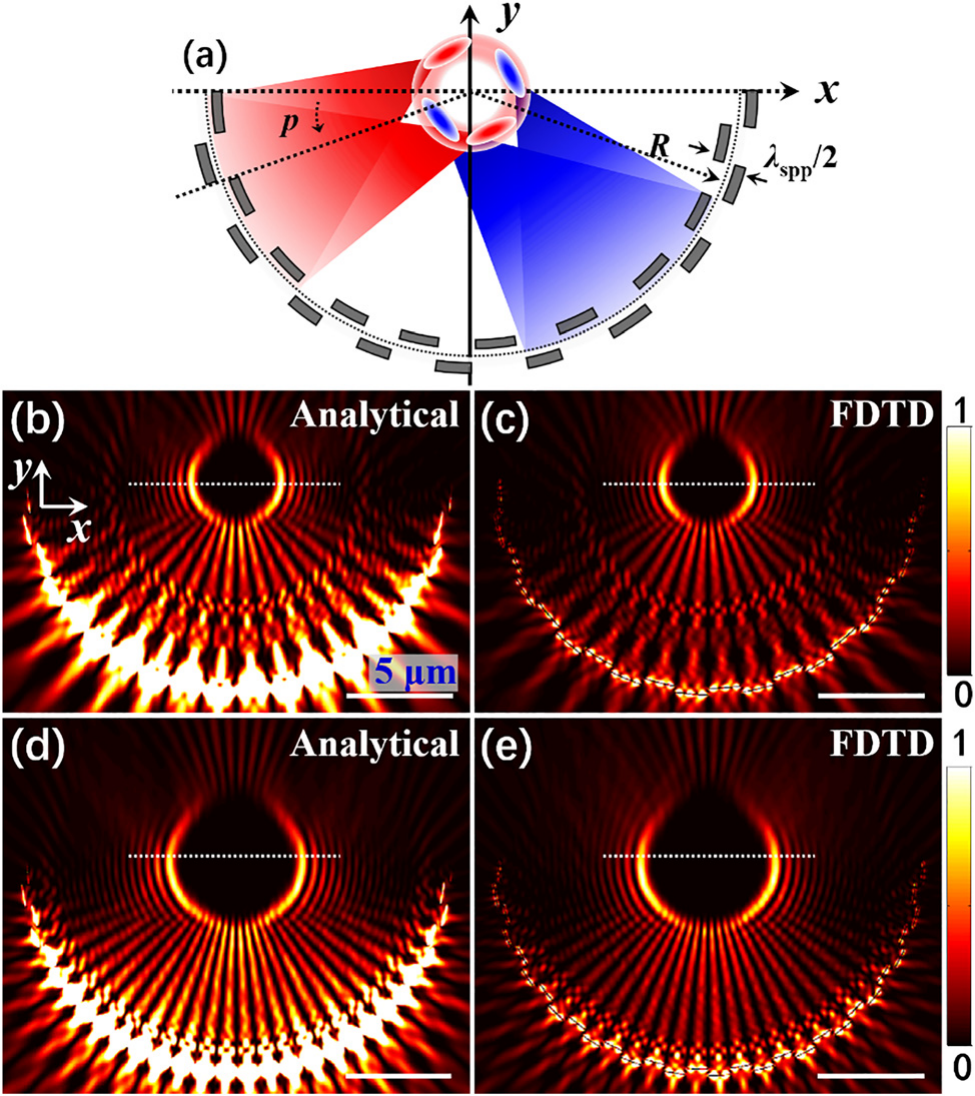
Generation of SPP bottle beam by proposed metalens. (a) Schematic diagram of the principle of synthesizing SPP bottle beam. The radius of the reference arc (black dotted curve line) is *R* = 10 μm, and the distance between two adjacent arc slits in the radial direction is *λ*_spp_/2. (b)–(e) Intensity distribution of the SPP bottle beam. (b) and (c) *α* = 180°, *p* = 18°, (d) and (e) *α* = 180°, *p* = 12°. (b) and (d) are analytical results. (c) and (e) are FDTD simulation results. All other parameters are as in Figure 1.

To further verify the universality of the proposed method in the generation of structured SPP field, next we produce an SPP bottle beam [48], [49], [50] and modulate its shape with the on-chip plasmonic metalens. Figure 3(a) shows the schematic diagram of the synthetic method of an SPP bottle beam. Here, the plasmonic metalens structure is composed of a series of arc-shaped double-layer slits arranged periodically along the angular direction. The results in Figure 1(d) and (e) prove that when the opening angle of the arc-shaped double-layer structure is small, a pattern of SPP dual-focus can be formed, because the small opening angle satisfies the paraxial approximation condition of FT, and the generated SPP focal field depends on the FT results as indicated in Figure 1(j) and (k). As the opening angle increases as shown in Figure 3(a), the whole structure can be equivalent to multiple arc-shaped double-layer structures with a small opening angle (Figure 1(d)), and the dual-focus generated by each double-layer structure distributes on a circle around the geometric center (as shown in red and blue regions of Figure 3(a)), and thus the superposition of these multiple dual-focus forms a ring-shaped SPP pattern, that is, the SPP bottle beam. The intensity distribution of the ***E***_**z**_ component of the SPP bottle beam calculated by Eq. (4) is shown in Figure 3(b) (*p* = 18°). We can observe that SPPs excited at different angles of the structure all propagate toward the center, and form a wheel-shaped field with the periodic bright and dark regions along the radial direction during the focusing process, which is actually the Talbot effect [51, 52] excited by the periodic slits. Then the wheel-shaped fields interfere in the central focal region to form an SPP bottle beam. In addition, when the period of the slits is decreased, the size of the bottle beam is increased as shown in Figure 3(d) (*p* = 12°), due to the same reason in Figure 1(k). The FDTD simulation results reproduce the above phenomenon very well under the same conditions (Figure 3(c) and (e)), verifying that the proposed method can generate and control complex SPP fields.

**Figure 4:**
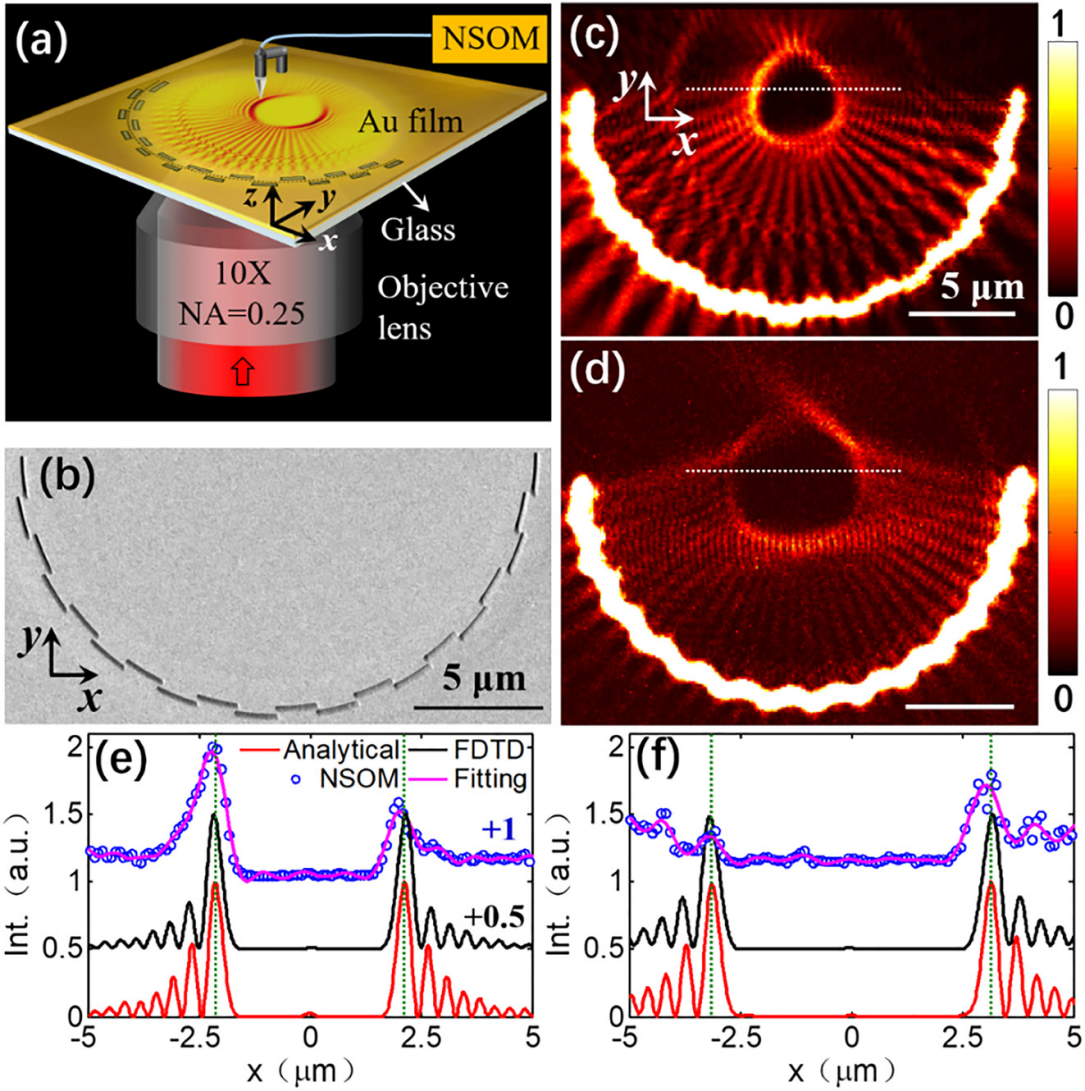
Experimental verification of SPP bottle beam. (a) Schematic diagram of the experimental system. The metalens structure is composed of a glass substrate (*n* = 1.515) and a gold film (200 nm in thickness) etched with arc-shaped slits (100 nm in width) on a glass substrate in the *xy* plane. A *y*-polarized incident light (*λ* = 632.8 nm) is focused by an objective lens (10×, NA = 0.25) and illuminated on the sample from the bottom. Then the SPP field distribution is detected by an NSOM. (b) SEM image of the metalens sample. (c) and (d) are experimental results with parameters: (c) *α* = 180°, *p* = 18°, (d) *α* = 180°, *p* = 12°. (e) and (f) Comparison of the normalized intensity distribution in the focal line (white dash lines in Figures 3, 4(c), and (d)) in the cases of analytical calculation, FDTD simulation, and NSOM measurement.

To verify the reliability of the proposed method for actual on-chip structures and devices, we take the SPP bottle beam as an example to carry out the experimental verification. As shown in Figure 4(a), the designed SPP bottle beam is experimentally measured by a near-field scanning optical microscope (NSOM), and the SEM image of the corresponding plasmonic metalens structure is shown in Figure 4(b). A *y*-polarized incident light (*λ* = 632.8 nm) is focused by an objective lens and illuminated on the sample from the bottom. The NSOM measured intensity distribution of the SPP bottle beam is shown in Figure 4(c) and (d), which agree well with the theoretical prediction in Figure 3. Furthermore, the normalized intensity distribution on the focal line (white dashed lines in Figure 4(c) and (d)) is extracted and compared with that of the analytical calculation (Figure 3(b) and (d)) and FDTD simulation results (Figure 3(c) and (e)), as shown in Figure 4(e) and (f). It can be clearly observed that the positions of the peak intensity of these curves are in good agreement, verifying the accuracy and effectiveness of the proposed method.

## 4 Conclusion

In this paper, we propose and verify a novel method for flexible generation of structured SPP field with on-chip plasmonic metalenses. The metalens is composed of multiple plasmonic focusing nanostructures whose focal shape and position can be independently manipulated, and through their superposition, SPP fields with specially designed patterns are obtained, including S-/W-shaped SPP focal fields and SPP bottle beams, and the effectiveness of the proposed method is verified experimentally. It is worth noting that, theoretically, this method is suitable not only for SPP but also for other on-chip propagating waves such as Bloch surface waves [53]. This work provides more degrees of freedom for on-chip photonics manipulation and has great application value in the fields of photonics information processing, on-chip optical computing, and integrated photonic circuits.
